# Transcriptomic Characterization of the Effects of Selenium on Maize Seedling Growth

**DOI:** 10.3389/fpls.2021.737029

**Published:** 2021-11-23

**Authors:** Lingling Dou, Zailong Tian, Qin Zhao, Mengting Xu, Yiran Zhu, Xiaoyue Luo, Xinxing Qiao, Rui Ren, Xianliang Zhang, Huaizhu Li

**Affiliations:** ^1^School of Chemistry and Chemical Engineering, Xianyang Normal University, Xianyang, China; ^2^College of Life Sciences, Shaanxi Normal University, Xi’an, China; ^3^Shaanxi Hygrogeology Engineering Geology and Environment Geology Survey Center, Xi’an, China; ^4^State Key Laboratory of Cotton Biology, Institute of Cotton Research, Chinese Academy of Agricultural Sciences, Anyang, China

**Keywords:** Selenium, maize seedlings, RNA-Seq, auxin, lignin, growth

## Abstract

Selenium (Se) is a trace mineral element in soils that can be beneficial to plants in small amounts. Although maize is among the most economically important crops, there are few reports on the effects of Se on maize seedling growth at the molecular level. In this study, the growth of maize seedlings treated with different concentrations of Na_2_SeO_3_ was investigated, and the physiological characteristics were measured. Compared with the control, a low Se concentration promoted seedling growth, whereas a high Se concentration inhibited it. To illustrate the transcriptional effects of Se on maize seedling growth, samples from control plants and those treated with low or high concentrations of Se were subjected to RNA sequencing. The differentially expressed gene (DEG) analysis revealed that there were 239 upregulated and 106 downregulated genes in the low Se treatment groups, while there were 845 upregulated and 1,686 downregulated DEGs in the high Se treatment groups. Both the Gene Ontology (GO) and the Kyoto Encyclopedia of Genes and Genomes (KEGG) annotation analyses showed a low concentration of the Se-stimulated expression of “DNA replication” and “glutathione (GSH) metabolism”-related genes. A high concentration of Se repressed the expression of auxin signal transduction and lignin biosynthesis-related genes. The real-time quantitative reverse transcription PCR (qRT-PCR) results showed that in the low Se treatment, “auxin signal transduction,” “DNA replication,” and lignin biosynthesis-related genes were upregulated 1.4- to 57.68-fold compared to the control, while, in the high Se concentration treatment, auxin signal transduction and lignin biosynthesis-related genes were downregulated 1.6- to 16.23-fold compared to the control. Based on these transcriptional differences and qRT-PCR validation, it was found that a low dosage of Se may promote maize seedling growth but becomes inhibitory to growth at higher concentrations. This study lays a foundation for the mechanisms underlying the effects of Se on maize seedling growth.

## Introduction

Selenium (Se) is a trace mineral element that is essential to humans and animals, which can be acquired by plants from the soil (Cao et al., 2018). There are five valence states of Se (i.e., Se^6+^, Se^4+^, Se^2+^, Se^0^, and Se^2–^); those that can be absorbed by plants are selenites (SeO_3_^2–^) and selenates (SeO_4_^2–^). Selenates and selenites are the two most widely used forms of inorganic Se (Li et al., 2008; Wang et al., 2019). The absorbed inorganic forms must be converted into organic forms such as selenocysteine (SeCys) before they can be further utilized by plants (Van Hoewyk, 2013). The effects of Se treatment on plant growth are highly dose-dependent: while low doses promote growth, higher doses have the opposite inhibitory effect.

Many studies have demonstrated that low-dose selenite supplements promote plant growth. For instance, the application of the low concentrations of both selenite and selenate increased wheat shoot and root biomass (Boldrin et al., 2016). The foliar application of selenite increased the Se content of peach fruit along with increasing flesh firmness and decreasing soluble solid content (Pezzarossa et al., 2012). Low Se concentrations stimulated primary root elongation and the number of lateral roots by triggering auxin biosynthesis and transport in tobacco (Jia et al., 2018). Se applied in low concentrations stimulated the growth of green alga *Chlorella pyrenoidosa* and also promoted the accumulation of photosynthetic pigments in the halophile green microalgae *Dunaliella salina* (Constantinescu-Aruxandei et al., 2019).

Antioxidant trials have shown that Se treatment increased the amounts of various phenolic compounds and their antioxidant activity in broccoli seedlings (Bachiega et al., 2016). The foliar application of Se in garden pea plants increased Se content, total polyphenols, and antioxidant capacity (Alžbeta et al., 2017). Both Se foliar application and Se fertilization increased Se content and total polyphenol content in tomatoes (Alena et al., 2019). Furthermore, the Se application is thought to promote crop yield and quality (Hossain et al., 2021). Other studies have shown that both the Se application and the overexpression of glutathione peroxidase (GSHPx) could protect human cells against UV-induced DNA mutations or chromosome breakage (Baliga et al., 2007).

Selenium can be a beneficial element for plants, and appropriate concentrations of selenite can promote the growth and development of plants as well as stress resistance. Se treatment was shown to protect oilseed rape leaves from pathogen infection by upregulating defense gene expression (Xu et al., 2020). The exogenous application of Se alleviated the detrimental effects of NaCl stress and enhanced photosynthesis in sunflowers (Ghawana, 2017).

High doses of Se were shown to decrease the dry root weight of wheat, relative to that of the controls (Li et al., 2008). The high doses of Se were also reported to repress growth in maize (Jiang et al., 2017), tea (Cao et al., 2018), and peach plants (Pezzarossa et al., 2012). The excessive application frequently results in detrimental Se phytotoxicity that inhibits plant growth.

Maize (*Zea mays* L.) is one of the most important food crops in the world and is also important for the livestock feed industry and biofuel production (Meng et al., 2016). The foliar applications of a range of Se concentrations revealed that a moderate Se (1 μM) treatment increased plant height and biomass in maize seedlings. Se application *via* fertigation was shown to increase pollen germination under H_2_O_2_-stress in maize seedlings (Del Pino et al., 2019); Se application significantly reduced cadmium (Cd) toxicity symptoms in maize (Zhang et al., 2019). While Se has clearly been demonstrated to be beneficial to plants at the physiological level, studies on the effects of Se treatment on maize have primarily focused on the physiological effects and total Se content in grains (Roberto et al., 2019), while the underlying molecular mechanisms of Se function in maize growth have largely gone unexamined.

In this study, several approaches were used to study the effect of selenite (Na_2_SeO_3_) on maize seedling growth. Maize seedlings were given exogenous applications of Se at a low concentration (≤ 1 μM Na_2_SeO_3_) and a high concentration (≥ 10 μM Na_2_SeO_3_), and growth was observed, revealing increased and decreased seedling growth, respectively. Gene expression changes resulting from Se treatment were determined by subjecting samples from maize seedlings treated with 0, 1, and 10 μM Na_2_SeO_3_ to RNA-sequencing (RNA-seq); bioinformatics and the real-time quantitative reverse transcription PCR (qRT-PCR) analysis revealed that Se may facilitate maize seedling growth by increasing the expression of DNA replication and glutathione (GSH) metabolism-related genes, while it may decrease maize growth by disrupting the expression of auxin signal transduction and lignin biosynthesis-related genes.

## Materials and Methods

### Plant Treatments

Fully formed and uniform maize (*Z. mays* L. cv. Shaan K818) seeds were selected and planted in plastic pots filled with clean sandy soil and vermiculite. The seedlings were placed in a cultivation chamber at 28°C (light)/22°C (dark), a 14-h light/10-h dark photoperiod, and the light intensity was kept at a constant 300 μmol⋅m^–2^⋅s^–1^ during the daytime. Half-strength Hoagland mixture (Liu et al., 2016) was dissolved into double-distilled water (ddH_2_O) to prepare the nutrient solution, to which Na_2_SeO_3_ was added to create treatment solutions with the Na_2_SeO_3_ concentrations of 0, 0.1, 1, 10, 20, and 30 mM, referred to as CK, T1, T2, T3, T4, and T5, respectively.

After the seedlings emerged to 2 cm, they were sprayed with the treatment solutions as described previously (Jiang et al., 2017). The CK, T1, T2, T3, T4, and T5 treatment solutions (200 ml) were sprayed on the maize seedlings to create the six treatment groups. The experiment included six treatments with three replicates each. Each replicate was composed of three pots of nine plants. The seedlings were treated three times every day and treated for 7 days in total (Supplementary Figure 1).

### Plant Fresh and Dry Weight Measurements

After the treatment, the shoots were separated from roots; the length and fresh weight of shoots and roots were measured immediately. The samples were divided into two parts: one was immediately frozen in liquid nitrogen and stored at −80°C, while the other was dried at 80°C to a constant weight.

### Determination of Total Selenium Concentration

The dried shoots and roots (0.1 g) were digested by HNO_3_-H_2_O_2_ according to the previously described methods, and the digested samples were diluted up to 20 ml with ddH_2_O (Luo et al., 2019). The total Se concentration was analyzed by inductively coupled plasma mass spectrometry (ICP-MS, Agilent 7900, Agilent Technologies, United States). Total Se standard solutions were prepared by diluting the corresponding stock solutions (Se standard 1,000 mg⋅L^–1^ for AAS TraceCERT Sigma, Italy) with ddH_2_O. The methods were validated with a recovery test (*n* = 3) as in the previous study (Marika et al., 2018) by adding Se standard solution (4 mg⋅L^–1^) into the mixture of the samples and HNO_3_ prior to digestion.

### RNA Extraction and Sequencing

The mRNA was extracted using the Omega Plant RNA Kit (Code No. R6827, Omega, United States), and the RNA content and quality were evaluated with the NanoDrop 2000 (Thermo, United States). Oligo(dT) magnetic beads were used for the mRNA enrichment of the samples. Transcriptome sequencing was performed on the Illumina HiSeq™ 2500 by Gene Denovo Biotechnology Co. (Guangzhou, China).

### Filtering of Clean Reads and Genome Alignment

The raw reads were further filtered by Fastq (version 0.18.0) to obtain clean reads following the following criteria: (1) adapter sequences, (2) reads containing more than 10% unknown nucleotides (N), (3) reads containing more than 50% of low quality (Chen et al., 2018), (4) and reads mapping to ribosome RNA (rRNA) database^1^ were removed by Bowtie 2 (Langmead and Salzberg, 2012). The dataset is available from the NCBI Short Read Archive (SRA) under accession No. PRJNA738971.

### Gene Abundance and Differentially Expressed Gene Analysis

Clean reads were mapped to the *Z. mays* genome (B73 version 4) (Jiao et al., 2017) using HISAT version 2.2.4 (Kim et al., 2015). The expression abundances of the mapped reads were evaluated and normalized by the fragment per kilobase of transcript per million mapped reads (FPKM) method using StringTie version 1.3.1 (Pertea et al., 2015, 2016).

The differentially expressed gene (DEG) analysis was performed using the DESeq2 (Love et al., 2014) software between two different groups and using edgeR between two samples (Robinson et al., 2010). Genes from the comparison groups, CK vs. T2 and CK vs. T3, having a false discovery rate (FDR) ≤ 0.05 and the absolute value of log2 (ratio of two FPKM values) ≥ 1, were considered as DEGs. To evaluate the repeatability between the replicates, the Pearson’s correlation coefficient values were calculated using the R software (Fukushima, 2013).

### Gene Ontology and Kyoto Encyclopedia of Genes and Genomes Annotation of Differentially Expressed Genes

The DEGs were queried against the Gene Ontology (GO) database^2^ using the Blast2GO program (Gotz et al., 2008). GO annotations with both *p-*value and FDR values ≤ 0.05 were considered to be significantly enriched GO terms. DEGs participating in metabolic pathways were analyzed by the Kyoto Encyclopedia of Genes and Genomes (KEGG) orthology-based annotation system (KOBAS 2.0), and the annotations with both *p-*value and FDR values < 0.05 were considered to be significantly enriched in the KEGG pathway (Kanehisa and Goto, 2000; Xie et al., 2011).

### qRT-PCR Experiments

A total of 100 ng of RNA was used for the first-strand cDNA synthesis using the EasyScript^®^ All-in-One First-Strand cDNA Synthesis SuperMix for qPCR Kit (Trans, China) according to the protocol of the manufacturer. Quantitative PCRs (qPCRs) were performed on the S1000™ Thermal Cycler (BioRad, United States) using PerfectStart™ Green qPCR SuperMix (Trans, China). The qPCR was conducted in 20 μl reaction volumes consisting of 2.0 μl cDNA, 1.0 μl forward and reverse primers (10 μM), 10 μl SYBR FAST 2 × PerfectStart™ Green qPCR SuperMix, and 7 μl ddH_2_O. The thermocycling conditions were as follows: pre-denaturation for 30 s at 95°C, followed by 40 cycles of 95°C for 5 s, 60°C for 15 s, and 72°C for 20 s. Three technical replicates were performed. The primers were designed using OLIGO 7 software (Rychlik, 2007) and are listed in Supplementary Table 1. The threshold cycle (Ct) values of all genes were normalized to the internal reference gene, *ZmActin* (NCBI accession No. NM_001155179) (Zhai et al., 2019); the relative gene expression level was calculated using the 2^–ΔΔCt^ method (Livak and Schmittgen, 2001).

### Statistical Analysis

All results were analyzed using a one-way ANOVA, and significantly different means between treatments were identified with a Tukey’s test at a 0.05 significance level, using SPSS 17.0 software (Norušis, 2008).

## Results

### Growth of Maize Seedlings Treated With Different Concentrations of Selenium

To investigate the effects of Se treatment on maize growth, different concentrations (i.e., 0, 0.1, 1, 10, 20, and 30 μM, represented as CK, T1, T2, T3, T4, and T5, respectively) were exogenously applied on maize seedlings.

**FIGURE 1:**
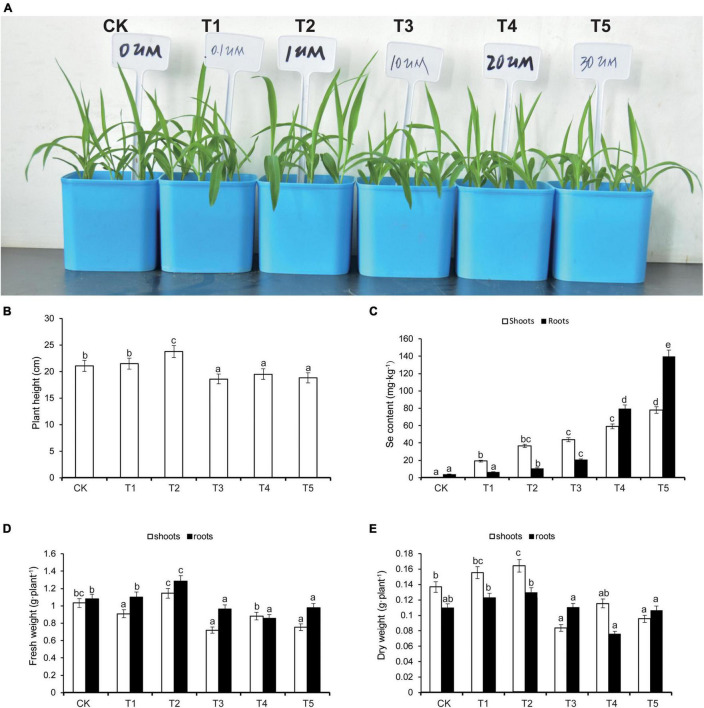
Effects of selenium (Se) treatment on plant growth **(A)**, plant height **(B)**, Se content **(C)**, fresh weight **(D)**, and dry weight **(E)** in the shoots and roots, respectively. Different treatments were added with or without different concentrations of Se. The abbreviations of CK, T1, T2, T3, T4, and T5 indicates 0 μM Na_2_SeO_3_, 0.1 μM Na_2_SeO_3_, 1 μM Na_2_SeO_3_, 10 μM Na_2_SeO_3_, 20 μM Na_2_SeO_3_, and 30 μM Na_2_SeO_3_, respectively. The statistical significance was determined using a one-way ANOVA combined with Tukey’s test. Columns labeled with different letters between treatments represent significant differences (*p* ≤ 0.05). Data are presented as mean ± SE (*n* = 27).

After 7 days of treatment, the heights of maize seedlings were significantly higher in the T2 group relative to CK, and in groups T3, T4, and T5, seedling heights were significantly lower than CK (Figures 1A,B). Higher concentrations of applied Na_2_SeO_3_ corresponded with higher total Se contents, both in shoots and roots of maize seedlings. The total Se contents in T2, T3, T4, and T5 were significantly higher than CK (Figure 1C). We further measured the fresh weight and dry weight, of both shoots and roots, under the five treatments. Both the dry weight and the fresh weight were heavier in T2 than CK; however, both the dry weight and the fresh weight were lighter in T3, T4, and T5 than CK (Figures 1D,E). Thus, we concluded that low concentration (1 μM) Na_2_SeO_3_ treatment of maize seedlings promoted their growth, whereas higher concentrations (10 μM) inhibited growth.

### Mapping and Quantitative Assessment of Illumina Sequencing Results

To determine the gene expression differences resulting from low Se concentration (T2, 1 μM) and high Se concentration (T3, 10 μM) treatments that may explain, respectively, the observed increases and decreases in plant growth, the root samples from the CK, T2, and T3 treatments were subjected to RNA-seq. In total, nine sequencing libraries, including three replicates of each treatment, labeled CK1 to CK3, T2-1 to T2-3, and T3-1 to T3-3, were constructed. The raw data were filtered, and high-quality (HQ) clean reads from the nine libraries ranged from 8,163,465,512 to 12,078,279,388 bp. From the HQ clean data, the Q20 and Q30 percentages were over 98.23 and 94.78%, respectively. The average GC content of the HQ clean reads was ∼58.65% (Table 1). The HQ clean reads of each library aligned well to the *Z. mays* genome (B73 version 4) (Jiao et al., 2017). The mapped proportion of the HQ clean reads in the nine transcriptomic libraries ranged from 83.26 to 86.41%, and the unique mapped reads accounted for 80.74 to 83.79% (Table 1). The mapped ratios and proportion of HQ reads indicated that the quality of the RNA-seq data was high.

**TABLE 1:** Number of reads sequenced and mapped to the maize genome.

Sample	CK1	CK2	CK3	T2-1	T2-2	T2-3	T3-1	T3-2	T3-3
CleanData (bp)	9,339,900,566	9,923,200,550	12,078,279,388	9,436,933,630	10,155,265,580	9,396,125,507	9,742,691,054	8,163,465,512	9,320,164,356
AF_Q20 (%)	9,186,607,575 (98.36%)	9,766,221,744 (98.42%)	11,861,462,136 (98.20%)	9,251,132,361 (98.03%)	9,973,189,532 (98.21%)	9,229,225,605 (98.22%)	9,560,296,259 (98.13%)	8,019,219,113 (98.23%)	9,149,398,988 (98.17%)
AF_Q30 (%)	8,882,987,966 (95.11%)	9,454,212,886 (95.27%)	11,444,410,685 (94.75%)	8,900,472,540 (94.32%)	9,623,099,188 (94.76%)	8,905,016,041 (94.77%)	9,214,826,308 (94.58%)	7,737,615,698 (94.78%)	8,821,254,922 (94.65%)
AF_GC (%)	5,480,578,738 (58.68%)	5,833,242,850 (58.78%)	7,091,529,710 (58.71%)	5,556,857,872 (58.88%)	5,906,108,784 (58.16%)	5,549,966,282 (59.07%)	5,728,205,838 (58.79%)	4,780,687,626 (58.56%)	5,422,474,596 (58.18%)
Raw reads	62,964,956	66,852,732	81,401,718	63,645,596	68,345,506	63,348,054	65,811,464	54,926,362	62,817,546
Clean reads (%)	62,846,162 (99.81%)	66,740,366 (99.83%)	81,245,912 (99.81%)	63,522,492 (99.81%)	68,212,716 (99.81%)	63,229,566 (99.81%)	65,661,702 (99.77%)	54,804,804 (99.78%)	62,689,336 (99.80%)
Unmapped ribosome Reads (%)	61,622,132 (98.05%)	66,305,270 (99.35%)	79,699,102 (98.10%)	62,015,772 (97.63%)	66,864,096 (98.02%)	61,468,390 (97.21%)	64,502,834 (98.24%)	53,900,700 (98.35%)	62,092,140 (99.05%)
Unmapped genome (%)	9,455,203 (15.34%)	9,715,191 (14.65%)	11,623,136 (14.58%)	8,478,020 (13.67%)	9,083,741 (13.59%)	9,004,428 (14.65%)	9,021,829 (13.99%)	7,604,951 (14.11%)	10,396,574 (16.74%)
Unique Mapped genome (%)	50,617,489 (82.14%)	54,908,412 (82.81%)	66,000,363 (82.81%)	51,965,626 (83.79%)	56,027,827 (83.79%)	50,930,249 (82.86%)	53,836,194 (83.46%)	44,887,465 (83.28%)	50,131,385 (80.74%)
Multiple Mapped (%)	1,549,440 (2.51%)	1,681,667 (2.54%)	2,075,603 (2.60%)	1,572,126 (2.54%)	1,752,528 (2.62%)	1,533,713 (2.50%)	1,644,811 (2.55%)	1,408,284 (2.61%)	1,564,181 (2.52%)
Total Mapped genome (%)	52,166,929 (84.66%)	56,590,079 (85.35%)	68,075,966 (85.42%)	53,537,752 (86.33%)	57,780,355 (86.41%)	52,463,962 (85.35%)	55,481,005 (86.01%)	46,295,749 (85.89%)	51,695,566 (83.26%)

### Identification and Validation of Differentially Expressed Genes

**FIGURE 2:**
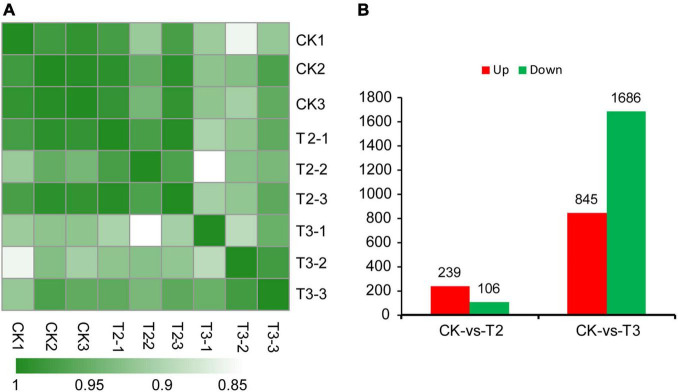
Heatmap of the Pearson’s correlation coefficients from comparisons between CK, T2, and T3 samples **(A)**. Differentially expressed genes (DEGs) in different comparison groups, i.e., CK vs. T2 and CK vs. T3 **(B)**.

To further analyze the reliability of the RNA-seq data, Pearson’s correlation coefficients were calculated. The biological replicates of the same treatment shared high correlation coefficients, ranging from 0.90 to 0.99 (Figure 2A). To illustrate the functions of Se in maize seedling growth, DEGs with both FDR values and *p*-values < 0.05 were identified. Compared with the control group (CK), maize seedlings in the T2 group (1 μM Na_2_SeO_3_) had 239 upregulated and 106 downregulated DEGs. There were 2,531 DEGs between CK and T3 (10 μM Na_2_SeO_3_); 845 DEGs were upregulated, and 1,686 DEGs were downregulated (Figure 2B). The DEG analysis showed that increasing Na_2_SeO_3_ concentration induced more DEGs.

### Gene Ontology Annotation of Differentially Expressed Genes

**FIGURE 3:**
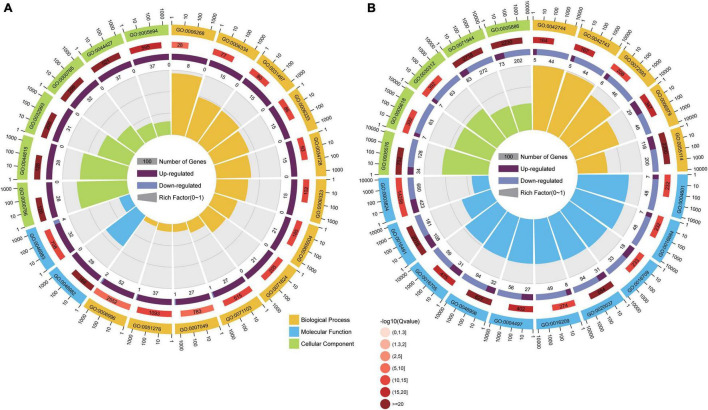
The analysis of the top 20 enriched Gene Ontology (GO) categories of DEGs in different comparison groups, i.e., CK vs. T2 **(A)** and CK vs. T3 **(B)**. The sectors indicate GO categories. The sector colors orange, blue, and green represent the biological process, molecular function, and cellular component, respectively. The three circles from inside to outside: the first circle is the number of DEGs enriched in the corresponding GO terms. Purple bars indicate upregulated gene numbers, and blue bars indicate downregulated gene numbers; the second circle is the number of background gene numbers in the corresponding GO terms; and the third circle is the enriched GO terms.

The GO terms with both *p*-values and corrected *p*-values ≤ 0.05 were considered significantly enriched. The enriched GO terms were classified into three categories, namely, biological process, molecular function, and cellular components. For DEGs in the comparison group CK vs. T2, there were 36, 22, and 83 significantly enriched GO terms in the cellular components, molecular functions, and biological process categories, respectively (Figure 3A and Supplementary Table 2). We further analyzed the top 20 GO enrichment categories of the DEGs from this comparison group, of which 12 were in the biological process category, including 3 GO terms involved in chromatin assembly (GO:0031497, GO:0006333, and GO:0051276), 2 involved in nucleosome assembly (GO:0034728 and GO:0006334), and 5 involved in the DNA replication process (GO:0006323, GO:0065004, GO:0071824, GO:0071103, and GO:0006268). The molecular function category was enriched for two GO terms (GO:0046983 and GO:0046982) involved in protein hetero-and-dimerization activity. The cellular component category was enriched for six terms, which involved chromosomes and the chromatin complex (GO:0005694 chromosome, GO:0044427 chromosomal part, GO:0000785 chromatin, GO:0032993 protein-DNA complex, GO:0044815 DNA packaging complex, and GO:0000786 nucleosome). There were more upregulated DEGs (CK vs. T3) in the top 20 enrichment GO terms (Figure 3A), which suggested that low Se concentration treatment can affect chromosome assembly and DNA replication in maize seedlings.

According to the GO enrichment analysis of DEGs from the CK vs. T3 comparison group, there were 20, 103, and 117 GO terms significantly enriched in the cellular components, molecular functions, and biological process categories, respectively (Supplementary Table 3). The analysis of the top 20 GO terms (Figure 3B) showed that 10 GO terms in the molecular function category were related to the oxidoreductase activity: the five GO terms in the biological process category were involved in the oxidation-reduction process, and the five GO terms of cellular component category functioned in the cell wall and extracellular regions. There were more downregulated DEGs (CK vs. T3) in the top 20 enriched GO terms (Figure 3B), suggesting that the oxidation-reduction process might be downregulated by high Se concentration treatment in maize seedlings.

### Kyoto Encyclopedia of Genes and Genomes Annotation of Differentially Expressed Genes

The KEGG pathway analysis was used to identify the biological functions of DEGs at the systems level. In this study, the KOBAS database was used to annotate the KEGG pathway of DEGs (Table 2 and Supplementary Table 4). According to the KEGG analysis, the DEGs in the comparison group CK vs. T2 were grouped into the category “replication and repair” corresponding to four second-category KEGG pathways, namely, DNA replication, mismatch repair, nucleotide excision repair, and homologous recombination. The DEGs in the comparison group CK vs. T3 were grouped into the plant hormone signal transduction (ko04075) and six biosynthesis pathways of secondary metabolites (Table 2 and Supplementary Table 5).

**TABLE 2:** Kyoto Encyclopedia of Genes and Genomes (KEGG) pathway enrichment analysis of differentially expressed genes (DEGs) in different comparison groups: CK vs. T2 and CK vs. T3.

KEGG ID	KEGG pathway name (CK vs. T2)	Gene NO.	Background NO.	*P*-value	Corrected *p*-value
ko03030	DNA replication	12 (26.09%)	65 (1.62%)	0	0
ko03430	Mismatch repair	7 (15.22%)	46 (1.14%)	0.000001	0.000012
ko03420	Nucleotide excision repair	7 (15.22%)	69 (1.72%)	0.00001	0.000128
ko03440	Homologous recombination	6 (13.04%)	61 (1.52%)	0.000055	0.000525

**KEGG ID**	**KEGG pathway name (CK vs. T3)**	**Gene NO.**	**Background NO.**	***P*-value**	**Corrected *p*-value**

ko04075	Plant hormone signal transduction	26 (7.62%)	217 (5.39%)	0.000211	0.00571
ko00940	Phenylpropanoid biosynthesis	47 (13.78%)	153 (3.8%)	0	0
ko00901	Indole alkaloid biosynthesis	8 (2.35%)	22 (0.55%)	0.000272	0.00714
ko00941	Flavonoid biosynthesis	8 (2.35%)	28 (0.7%)	0.001681	0.019617
ko00950	Isoquinoline alkaloid biosynthesis	5 (1.47%)	14 (0.35%)	0.004475	0.039156
ko00902	Monoterpenoid biosynthesis	4 (1.17%)	7 (0.17%)	0.001444	0.018957
ko00909	Sesquiterpenoid and triterpenoid biosynthesis	4 (1.17%)	7 (0.17%)	0.001444	0.018957

### Low Selenium Concentration Treatment May Promote DNA Replication and Repair in Maize Seedlings

**FIGURE 4:**
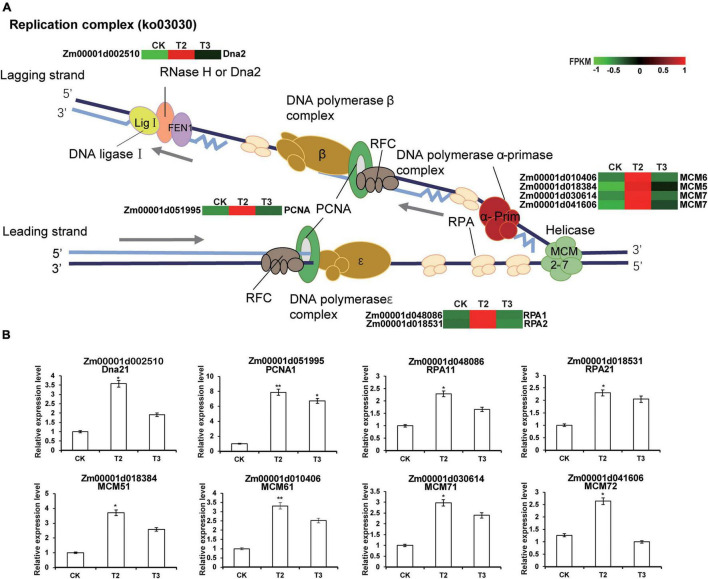
Transcriptional changes of DEGs involved in the DNA replication process in maize seedlings. **(A)** The heatmap was constructed using the fragment per kilobase of transcript per million mapped reads (FPKM) using online heatmap software (https://www.omicshare.com/tools/Home/Soft/heatmap). For the heatmaps, the transition from green to red represents the expression levels from low to high abundance. **(B)** DEGs involved in the DNA replication process are verified by real-time quantitative reverse transcription PCR (qRT-PCR). The statistical significance was determined using SPSS 16.0 with a one-way analysis of Tukey’s test. * represents *p* ≤ 0.05; ** represents *p* ≤ 0.01. The x-axis represents Se application treatments (CK, T2, and T3), and the y-axis indicates the relative expression levels. Error bar shows the SEs of three biological replicates.

Several previous studies have reported that Se is beneficial to plant growth and health and examined its effects at the physiological level (Jiang et al., 2017). However, few studies have investigated the effects of Se on plant growth at the gene transcriptional level. During DNA replication, the minichromosome maintenance protein (MCM), which is a DNA helicase, unwinds the double-stranded DNA (dsDNA) to produce the single-stranded DNA (ssDNA) (Wei and Zhao, 2016). Replication protein A (RPA) quickly binds newly generated ssDNA to protect it from damage and prevent the formation of secondary structures, which helps to ensure normal and orderly DNA replication (Marechal and Zou, 2015). During replication, a ringed structure of proliferating cell nuclear antigen (PCNA) wraps around the chromosome and recruits other proteins to efficiently copy the parent DNA. The stable binding of PCNA to DNA makes it an important platform for many other replication proteins (Liu et al., 2020). Dna2 is a nuclease-helicase, which functions in DNA end-resection during dsDNA break repair, Okazaki fragment processing, and the recovery of stalled DNA replication forks (RFs) (Appanah et al., 2020). Four *MCM* genes, two *RPA* genes, and both *PCNA* and *Dna2* genes were upregulated under low Se concentration (1 μM Na_2_SeO_3_) treatment but conversely downregulated under high Se concentration (10 μM Na_2_SeO_3_), and these genes were significantly enriched in DNA replication and repair (Figure 4). Additionally, other studies have shown that MCM, PCNA, and RPA proteins have roles in homologous recombination, nucleotide excision repair, and mismatch repair (Soniat et al., 2019). Given these findings, we speculated that low Na_2_SeO_3_ concentration treatment promotes maize seedling growth by enhancing DNA replication and repair processes to ensure accurate DNA replication.

### Differentially Expressed Genes Involved in Glutathione Metabolism

**FIGURE 5:**
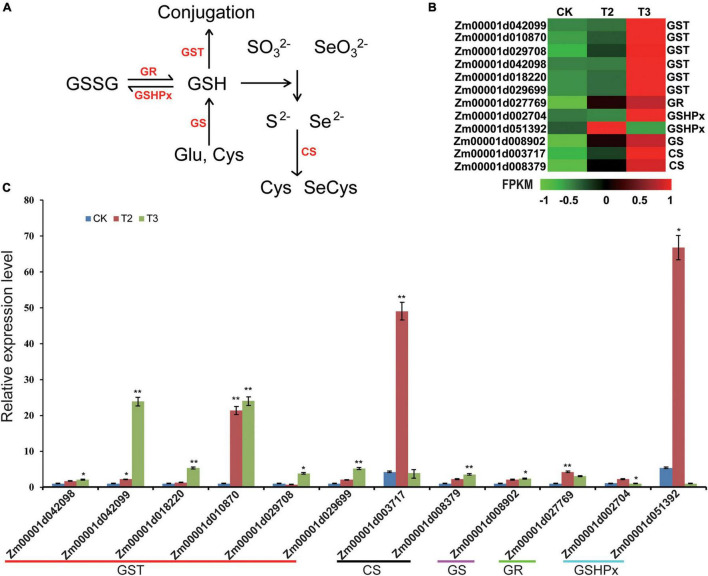
Expression profiles of DEGs involved in the transformation of inorganic Se into organic Se and glutathione (GSH) metabolism. **(A)** GSH and Se (S) metabolism process. Arrows represent the activation processes. GSSG, glutathione disulfide; GSH, glutathione; GR, glutathione reductase; GSHPx, glutathione peroxidase; GST, glutathione S-transferase; GS, glutathione synthetase; and CS, cysteine synthase. **(B)** The heatmap was constructed using the FPKM. For the heatmaps, the color from green to red represents low to high expression levels. **(C)** DEGs involved in GSH and Se (S) metabolism process are verified by qRT-PCR. The statistical significance was determined using SPSS 16.0 with a one-way analysis of Tukey’s test. * represents *p* ≤ 0.05; ** represents *p* < 0.01. The x-axis represents Se application treatments (CK, T2, and T3), and the y-axis indicates the relative expression levels. Error bar shows the SEs of three biological replicates.

Selenium and sulfur (S) are the members of the same group of metalloids in the periodic table, having similar ionic radii; therefore, the physicochemical properties of both elements are similar to one another (Bodnar et al., 2012). For Se or S to be metabolized into organic components, SO_3_^2–^ and SeO_3_^2–^ are reduced to Se^2–^ and S^2–^, a reaction catalyzed by GSH as the electron donor. Under the action of cysteine synthase (CS), S^2–^ and Se^2–^ could react with *O*-Acetylserine (OAS) to form cysteine (Cys) or Se-cysteine (SeCys) (Figure 5A). Cys or SeCys is the first step in the transformation of inorganic S or Se into other organic species (Zhou et al., 2020). Both RNA-seq and qRT-PCR (Figures 5B,C) experiments showed that, in correspondence with increasing SeO_3_^2–^ concentration, *CS* expression gradually increased, suggesting that Se promoted Cys or SeCys synthesis in maize seedlings.

Glutathione synthetase (GS) promoted the combination of Glu and Cys to produce GSH. GSH has the ability to scavenge reactive oxygen species (ROS), by GSHPx catalyzing GSH oxidized as glutathione disulfide (GSSG) by removing H_2_O_2_; glutathione S-transferase (GST) catalyzes the conjugation of GSH to electrophilic sites (Ferguson et al., 2012; Kou et al., 2019). GSSG could be reduced by glutathione reductase (GR) as GSH (Figure 5A). The expression analysis showed that the exogenous application of Se could increase the expression of GSH metabolism-related genes such as *GS*, *GSHPx*, *GR*, and *GST*. Above all, the expression analysis showed that the exogenously applied SeO_3_^2^ may lead to increased GSH metabolism in maize seedlings.

### High-Dose Selenium Treatment Inhibited the Expression of Auxin Signal Transduction-Related Genes in Maize Seedlings

**FIGURE 6:**
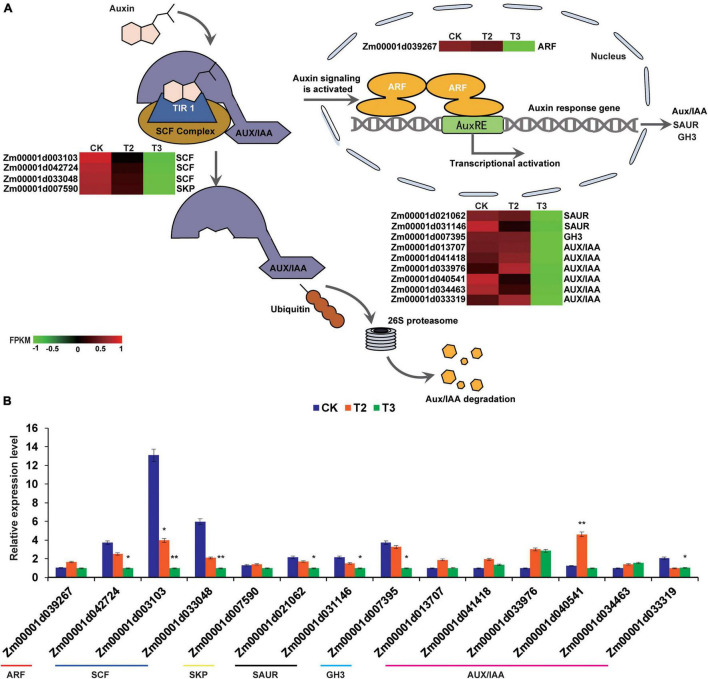
Expression profiles of auxin signaling transduction-related genes in CK, T2, and T3 samples. **(A)** Schematic diagram of auxin signal transduction and RNA-seq expression profiles. Auxin molecules recruit AUXIN/INDOLE ACETIC ACID (Aux/IAA) and transport inhibitor response (TIR) and bind with the SKP-Cullin-F-box (SCF) complex; Aux/IAA is subsequently labeled with ubiquitin, as well as recognized and degraded by the 26 S proteasome (Dharmasiri and Estelle, 2004). The heatmap was constructed using the FPKM, and the color from green to red represents the gene expression levels from low to high. **(B)** DEGs involved in auxin signal transduction are verified by qRT-PCR. The statistical significance was determined using SPSS 16.0 with a one-way analysis of Tukey’s test. * represents *p* ≤ 0.05; ** represents *p* ≤ 0.01. The x-axis represents Se application treatments (CK, T2, and T3), and the y-axis indicates the relative expression levels. Error bar shows the SEs of three biological replicates.

Auxin is one of the most important phytohormones, having important roles in regulating cell elongation (Leskovi et al., 2020). The comparison of CK with T3 (10 μM Na_2_SeO_3_) showed that DEGs were enriched in the auxin signal transduction process (Figure 6). During auxin signal transduction, auxin molecules bind to the receptor transport inhibitor response 1 (TIR1), and the conformational change of TIR1 enhances the tight binding of the SCF^*TIR*1^ [S-phase kinase associated protein (SKP)-Cullin-F-box (SCF), TRANSPORT INHIBITOR RESPONSE 1/AUXIN SIGNALING F-BOX (TIR1/AFB), and AUXIN/INDOLE ACETIC ACID (Aux/IAA)] complex to Aux/IAA. This promotes the degradation of the Aux/IAA protein by the 26S proteasome, thereby releasing auxin response factor (ARF), which forms a homodimer to promote the transcription of downstream genes, thus enabling the auxin reaction to proceed smoothly (Hager, 2003; Dharmasiri and Estelle, 2004). There were one *ARF*, three *SCF*, one *SKP*, six *Aux/IAA*, two *Small auxin up RNA* (*SAUR*), and one *Gretchen hagen 3* (*GH3*) genes downregulated under the high Se concentration treatment (T3) compared with CK (Figure 6). Based on the relative gene expression values between the groups of CK vs. T3 and CK vs. T2, we observed that the high-dose Se treatment downregulated auxin signal transduction process-related genes such as *ARF*, *Aux/IAA*, *SCF*, *SKP*, *SAUR*, and *GH3* genes.

### High-Dose Selenium Treatment Inhibited the Lignin Biosynthesis-Related Gene Expression in Maize Seedlings

Previous studies reported that Se could influence plant primary and secondary metabolism to enhance resistance to biotic and abiotic stresses and improve growth (Handa et al., 2018). The KEGG analysis showed that DEGs in the comparison group CK vs. T3 were significantly enriched in six secondary metabolism pathways and involved four secondary metabolites, namely, phenylpropanoid (PP), indole alkaloid, flavonoid, and terpenoid (Table 2).

**FIGURE 7:**
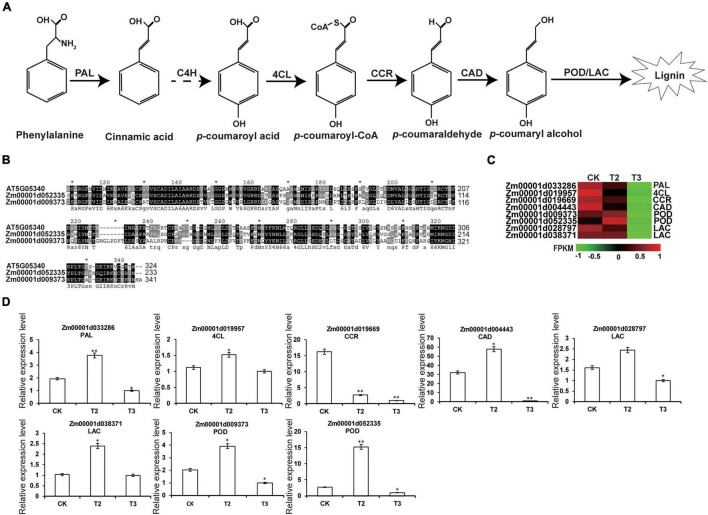
Expression profiling of DEGs related to lignin biosynthesis. **(A)** Biosynthesis pathway of lignin (Boerjan et al., 2003). Solid arrows and dotted arrows represent known and undiscovered parts of the pathway. PAL, phenylalanine ammonia-lyase; C4H, cinnamate 4-hydroxylase; 4CL, 4-coumarate-CoA ligase; CCR, cinnamoyl-CoA reductase; CAD, cinnamyl alcohol dehydrogenase; POD, peroxidase; and LCC, laccase. **(B)** Multiple sequences alignment of AtPOD (At5G05340) and ZmPOD (Zm00001d052335 and Zm00001d009373). **(C)** Expression heatmaps for DEGs (CK, T2, and T3) involved in lignin biosynthesis. The heatmap was constructed using the FPKM, and the color from green to red represents the gene expression levels from low to high. **(D)** DEGs involved in lignin biosynthesis are verified by qRT-PCR. The statistical significance was determined using SPSS 16.0 with a one-way analysis of Tukey’s test. * represents *p* ≤ 0.05; ^**^ represents *p* ≤ 0.01. The x-axis represents Se application treatments (CK, T2, and T3), and the y-axis indicates the relative expression levels. Error bar shows the SEs of three biological replicates.

Further analysis of the secondary metabolism pathways identified a successive lignin biosynthesis pathway with phenylpropionic acid as the starting point (Tan et al., 2013). Phenylalanine was successively catalyzed by phenylalanine ammonia-lyase (PAL), cinnamate 4-hydroxylase (C4H), 4-coumarate-CoA ligase (4CL), cinnamoyl-CoA reductase (CCR), cinnamyl-alcohol dehydrogenase (CAD), lignin peroxidase (POD), or laccase (LAC) to form lignin (Figure 7A; Boerjan et al., 2003). *AT5G05340* encodes a peroxidase involved in lignin biosynthesis (Hoffmann et al., 2020); however, Zm00001d052335 and Zm00001d009373 share the similarities of 56 and 51% with AT5G05340, respectively (Figure 7B). According to the RNA-seq and qRT-PCR expression patterns, successive genes involved in lignin biosyntheses such as *PAL*, *4CL*, *CCR*, *CAD*, *POD*, and *LAC* were highly expressed in T2 and showed low expression in T3, relative to CK (Figures 7C,D). Overall, the expression analysis of genes involved in lignin biosynthesis showed that the high dose of Na_2_SeO_3_ inhibited lignin biosynthesis-related gene expression.

## Discussion

In this study, our culture experiments showed that a low dose of Se enhanced maize seedling growth, but high doses suppressed it. To illustrate the transcriptional effects of Se on maize seedling growth, samples from control-, low Se concentration-, and high Se concentration-treated maize seedlings were used to perform RNA-seq experiments. Both the GO and the KEGG annotation analyses revealed that DEGs were involved in the DNA replication and the process categories of secondary metabolites. The qRT-PCR expression analysis was used to validate RNA-seq results, and the relative gene expression results from both revealed that the low Se concentration treatment may promote maize seedling growth by promoting DNA replication and GSH metabolite processes; conversely, the high Se concentration treatment inhibited maize seedling growth, potentially by decreasing the expression of auxin signal transduction and lignin biosynthesis-related genes.

### Selenium Active Functions in Maize Seedlings Growth: DNA Replication and Glutathione Metabolism

Studies on Se promoting plant growth have previously focused on the physiological effects; however, studies in humans and animals have shown that Se has vital functions in the DNA replication and repair processes. Soil application of Se increased shoot dry weight and grain yield of purple-grained wheat (Xia et al., 2020). Through the supplementation of tissue culture media, it was shown that animal or human diets with moderate levels of certain Se compounds could protect against the formation of DNA adducts, DNA or chromosome breakage, and chromosome gain or loss. The protective effects on mitochondrial DNA and on telomere length and function have also been observed (Ferguson et al., 2012). Additionally, in studies of human cancers, Se supplementation could reduce the frequency of DNA adducts and chromosome breaks, consequentially reducing the detrimental mutations that ultimately contribute to carcinogenesis (Bera et al., 2013). Studies demonstrated that the modification of Se-atom could significantly inhibit the non-specific DNA polymerization-induced mispriming (Hu et al., 2019), and the 2-Se-T atom with weak hydrogen-bonding ability can largely increase mismatch discriminations, maintaining the native DNA base pair (T/A) (Hassan et al., 2010).

Both Se and S have similar ionic radii and share similar physicochemical properties (Bodnar et al., 2012). Cys and SeCys are the end products of the assimilatory sulfate and selenite reduction pathways and serve as the unique donors of reduced S and Se for many Se- and S-containing compounds. Cys promotes the synthesis of GSH, a universal scavenger of ROS in plants (Speiser et al., 2018). The biological activity of Se begins with SeCys, which functions as a cofactor for the reduction of antioxidant enzymes, GSHPx (Ferguson et al., 2012). Both RNA-seq and qRT-PCR data revealed that Se treatment promoted the expression of Se and GSH metabolism-related genes in maize seedlings.

### Inhibitory Effects of Selenium on Maize Seedling Growth: Auxin Signal Transduction and Lignin Biosynthesis

Auxin is one of the most important phytohormones in the promotion of plant cell elongation (Dharmasiri and Estelle, 2004). Growth inhibition resulting from high Se concentration treatment is potentially related to the repression of auxin signal transduction (Pandey and Gupta, 2015). The applications of high concentrations of selenomethionine (SeMet) to rice seedlings inhibited the elongation of shoot and root length and decreased biomass compared with the control (Malheiros et al., 2020). Furthermore, auxin biosynthesis-related genes and IAA concentration were downregulated by SeMet treatment (Malheiros et al., 2020). Selenite treatment also downregulated the auxin efflux carrier, i.e., *PIN-formed 1* (*PIN1*) gene, while upregulating the auxin conjugator, i.e., *indol-3-acetate*β*-glucosyltransferase* gene in *Arabidopsis thaliana* (Lehotai et al., 2012). Moreover, in Se-treated *Arabidopsis*, the expression levels of auxin-related genes were associated with decreased root and shoot lengths (Van Hoewyk et al., 2008). A high concentration of Se can damage ryegrass growth by ROS accumulation, inhibiting plant growth (Hartikainen et al., 2000). In this study, the auxin signal-related genes such as *ARF*, *SCF*, *SAUR*, *GH3*, and *Aux/IAA* were downregulated.

Selenium may participate in the regulation of maize lignin biosynthesis. The rhizomes of *Atractylodes macrocephala* showed a significant negative correlation between insect attack rate and function of Se content (Zhou et al., 2021). Treatment with Se led to decreased growth in *A. thaliana* and repressed transcription of genes associated with the cell wall, such as *expansin* (*EXPL1*, *EXPA1*, and *EXPA8*) and *xyloglucan endotransglucosylase* (*XTHs*) genes (Ribeiro et al., 2016). Lignins are the phenolic polymers that work to thicken cell walls, thereby increasing resistance to pathogen attack (Russell et al., 2006). Peroxidase catalyzes PPs to form phenoxyl radicals and subsequently non-enzymatically extends the polymerization to form natural lignins (Korkina, 2007). Rice treated with Se had increased lignin content and thickness of the cell walls to improve the force of the cell walls, with the increased expression of lignin biosynthesis-related genes (i.e., *OsPAL* and *Os4CL3*) (Cui et al., 2018). The transcriptomic analysis showed that Se treatment-induced DEGs significantly enriched in the cell wall category in *Puccinellia distans* (Kok et al., 2020).

## Conclusion

**FIGURE 8:**
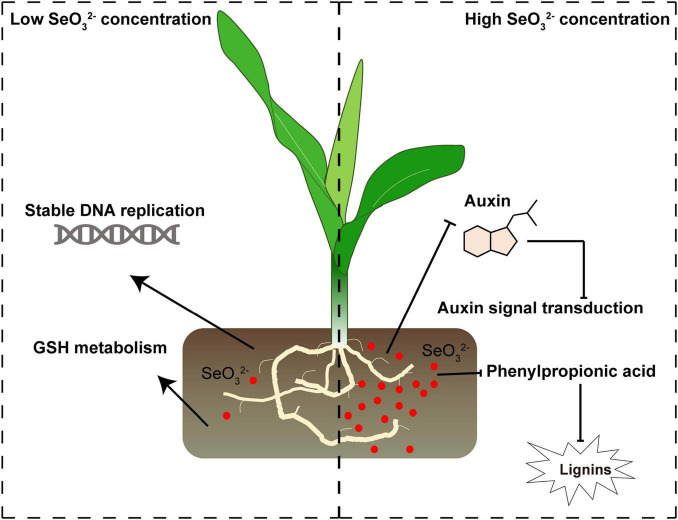
The schematic diagram for the mechanisms of low and high Se treatment effects on maize seedling growth. Se potentially promotes maize seedling growth by stabilizing DNA replication and GSH metabolism; the high doses of Se may have inhibited auxin signal transduction and lignin biosynthesis, reducing maize growth. Red circles represent SeO_3_^2–^. Arrows and inhibition lines represent the activation and suppression processes, respectively.

In this study, we illustrated the effects of different concentrations of Se treatment on gene expression in maize seedlings (Figure 8). We found that a low dose of Se (T2) enhanced maize seedling growth, whereas a high dose of Se (T3) suppressed growth. Bioinformatics and expression analysis showed that plant growth was potentially enhanced through the stabilization of DNA replication and GSH metabolism; growth may have been suppressed by inhibiting auxin signal transduction and phenylpropionic acid-mediated lignin biosynthesis. In the future, more studies will be required to characterize the molecular mechanism of Se-mediated maize development. Our study provides a molecular basis for uncovering Se functions in maize seedling growth.

## Data Availability Statement

The datasets presented in this study can be found in online repositories. The names of the repository/repositories and accession number(s) can be found in the article/Supplementary Material.

## Author Contributions

LD: conceptualization, methodology, software, and writing–original draft. ZT: software and methodology. QZ: data curation. MX: investigation and software. YZ: visualization and validation. XL: investigation and validation. XQ: investigation. RR and XZ: supervision. HL: writing, review and editing, and supervision. All authors contributed to the article and approved the submitted version.

## Conflict of Interest

The authors declare that the research was conducted in the absence of any commercial or financial relationships that could be construed as a potential conflict of interest.

## Publisher’s Note

All claims expressed in this article are solely those of the authors and do not necessarily represent those of their affiliated organizations, or those of the publisher, the editors and the reviewers. Any product that may be evaluated in this article, or claim that may be made by its manufacturer, is not guaranteed or endorsed by the publisher.

## Footnotes

1
http://www.plantrdnadatabase.com

2
http://www.geneontology.org

## Supplementary Material

Supplementary Figure 1 A flowchart for plant treatments.
