# Supplementary material for: Transcriptomic Characterization of the Effects of Selenium on Maize Seedling Growth
Source: Front Plant Sci. 2021 Nov 23;12:737029. doi: 10.3389/fpls.2021.737029 (PMC8650135; doi:10.3389/fpls.2021.737029)
Supplement: Supplementary Figure 1 — A flowchart for plant treatments. [file Image_1.pdf]

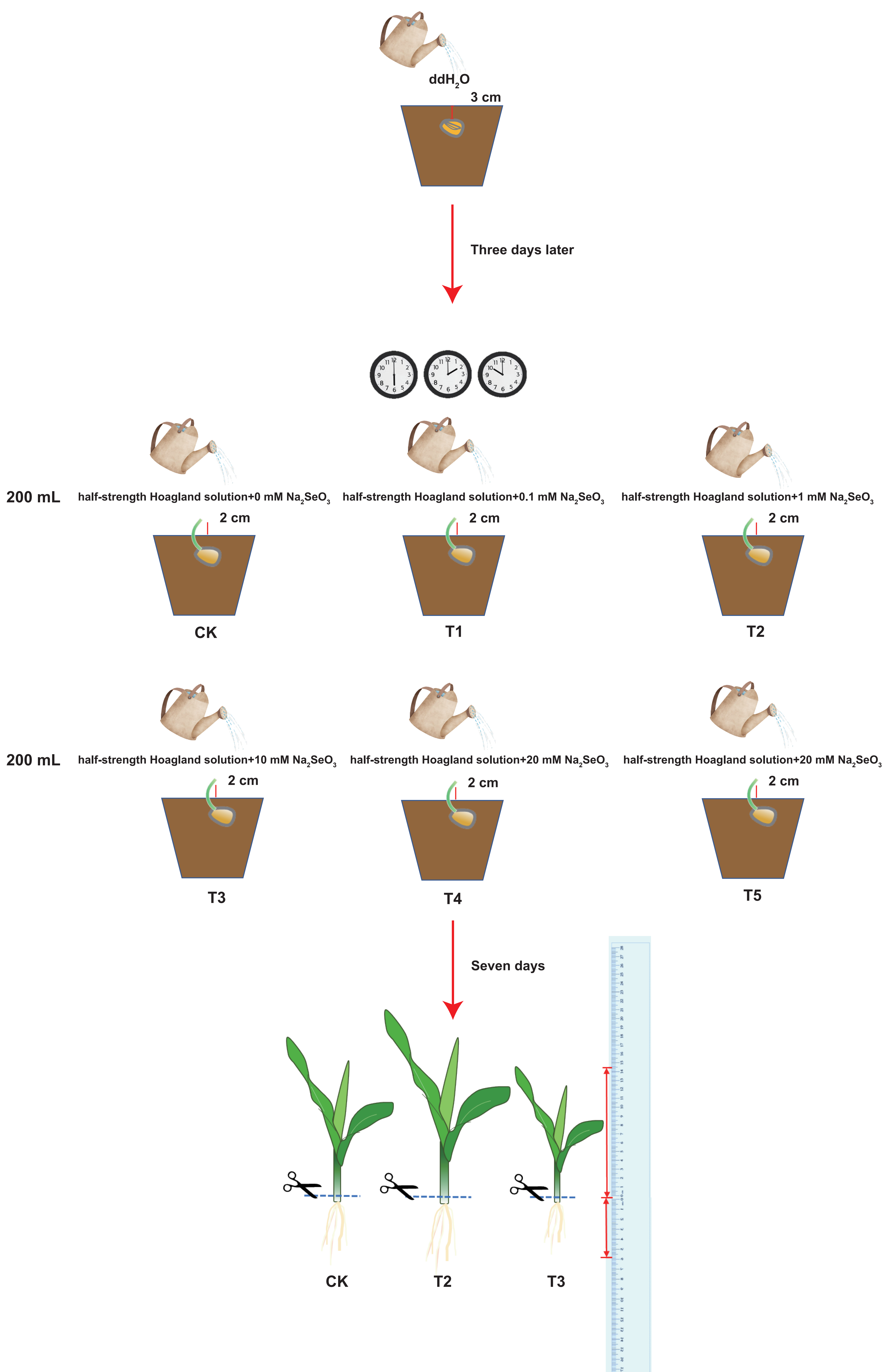

ddH2O
3 cm
Three days later
200 mL
half-strength Hoagland solution+0 mM Na2SeO3
half-strength Hoagland solution+0.1 mM Na2SeO3
half-strength Hoagland solution+1 mM Na2SeO3
2 cm
2 cm
2 cm
CK
T1
T2
200 mL
half-strength Hoagland solution+10 mM Na2SeO3
half-strength Hoagland solution+20 mM Na2SeO3
half-strength Hoagland solution+20 mM Na2SeO3
2 cm
2 cm
2 cm
T3
T4
T5
Seven days
CK
T2
T3
